# Supplementation of T_**3**_ Recovers Hypothyroid Rat Liver Cells from Oxidatively Damaged Inner Mitochondrial Membrane Leading to Apoptosis

**DOI:** 10.1155/2014/590897

**Published:** 2014-05-28

**Authors:** Sutapa Mukherjee, Luna Samanta, Anita Roy, Shravani Bhanja, Gagan B. N. Chainy

**Affiliations:** ^1^Department of Zoology, Utkal University, Bhubaneswar, Odisha 751004, India; ^2^Department of Zoology, Visva-Bharati (A Central University), Santiniketan, West Bengal 731235, India; ^3^Department of Zoology, Ravenshaw University, Cuttack, Odisha 753003, India; ^4^Department of Biotechnology, Utkal University, Bhubaneswar, Odisha 751004, India

## Abstract

Hypothyroidism is a growing medical concern. There are conflicting reports regarding the mechanism of oxidative stress in hypothyroidism. Mitochondrial oxidative stress is pivotal to thyroid dysfunction. The present study aimed to delineate the effects of hepatic inner mitochondrial membrane dysfunction as a consequence of 6-n-propyl-2-thiouracil-induced hypothyroidism in rats. Increased oxidative stress predominance in the submitochondrial particles (SMP) and altered antioxidant defenses in the mitochondrial matrix fraction correlated with hepatocyte apoptosis. In order to check whether the effects caused by hypothyroidism are reversed by T_3_, the above parameters were evaluated in a subset of T_3_-treated hypothyroid rats. Complex I activity was inhibited in hypothyroid SMP, whereas T_3_ supplementation upregulated electron transport chain complexes. Higher mitochondrial H_2_O_2_ levels in hypothyroidism due to reduced matrix GPx activity culminated in severe oxidative damage to membrane lipids. SMP and matrix proteins were stabilised in hypothyroidism but exhibited increased carbonylation after T_3_ administration. Glutathione content was higher in both. Hepatocyte apoptosis was evident in hypothyroid liver sections; T_3_ administration, on the other hand, exerted antiapoptotic and proproliferative effects. Hence, thyroid hormone level critically regulates functional integrity of hepatic mitochondria; hypothyroidism injures mitochondrial membrane lipids leading to hepatocyte apoptosis, which is substantially recovered upon T_3_ supplementation.

## 1. Introduction

Mitochondria, the powerhouses of eukaryotic cells, are instrumental in regulating the various signaling pathways involved in cell survival and death. The electron transport chain, localized in the inner membrane of mitochondrion, is the major source of intracellular reactive oxygen species (ROS), which renders the mitochondrion itself a highly vulnerable target [1, 2]. The mitochondrial oxidative stress has long been implicated in the aging process and in the pathogenesis of several human diseases, including cancer, neurodegenerative disorders, and thyroid dysfunction.

Thyroid hormones have profound impact upon mitochondrial biogenesis and activity [3, 4]. Hypothyroidism is known to diminish oxygen consumption and promote low metabolism causing disturbances in hemodynamic, cardiac, and renal functions [5]. The electron transport chain complexes and F_o_F_1_-ATPase activities are decreased in many tissues in thyroid deficient condition [6]. In fact, hypothyroid status was proposed as useful model to understand the molecular mechanisms involved in ischemia reperfusion, regulation of vascular function, and intravascular metabolism of lipoproteins [5]. Liver is well recognised as a major target organ for thyroid hormone [7]. In this perspective, studies on hepatic mitochondrial metabolism and functional integrity in thyroid dysfunction attain utmost relevance.

Previous reports have established significant modulation of various aspects of ROS metabolism and antioxidant defenses in the liver by experimentally induced hypo- and hyperthyroidism [8–10]. However, these reports were confined to whole mitochondrial fraction isolated from rat liver. We earlier delineated the mitochondrial effects, in particular, by reporting the influence of acute treatment of euthyroid rats with 3,5,3′-triiodo-L-thyronine (T_3_) upon hepatic ROS generation, glutathione (GSH) redox status, and protein oxidation following time kinetics, after subfractionating mitochondria into submitochondrial particles (SMP) and matrix fraction [11]. As an extension to this, we presently elucidate the effect of 6-n-propyl-2-thiouracil- (PTU-) induced hypothyroidism upon various relevant prooxidative and antioxidative parameters in adult rat liver SMP and mitochondrial matrix fraction along with histological and apoptosis analyses. In order to check whether the effects caused by hypothyroidism are reversed by T_3_, the above parameters were evaluated in a subset of hypothyroid rats administered with T_3_. Following similar experimental regimen, we earlier found that hypothyroid state inflicted changes in intramitochondrial protein and nonprotein thiol redox status in rat testes that could not be fully reversed when T_3_ was given to the hypothyroid rats [12]. It is opined that the present investigation in the light of our above-mentioned reports would enable comprehensive understanding as to how these important oxidative stress parameters are regulated in distinctive hepatic mitochondrial compartments in hypothyroidism, as to whether these are reversed by T_3_, and, if yes, as to what extent the damage is ameliorated.

## 2. Materials and Methods

### 2.1. Animals and Treatments

In the present experiment, liver tissues from rats of our earlier reported study were used [12]. The treatment regimen of the investigation is reported in brief. Six-month-old adult male Wistar rats (*Rattus norvegicus*) were obtained from the National Institute of Nutrition (Hyderabad, India) and housed in the Animal Room of the department at 25 ± 1°C. Animals were randomly assorted into three groups, each containing five animals. Group I (euthyroid) rats were fed with standard diet and drinking water* ad libitum.* Group II (hypothyroid) rats were treated with 0.05% (w/v) PTU in drinking water for six weeks. After completion of this time period, administration of PTU was stopped and rats were given one daily injection of 0.01 N NaOH, i.p. (vehicle for T_3_), for three consecutive days. Group III (hypothyroid+T_3_) rats were treated with 0.05% (w/v) PTU in drinking water for six weeks. After completion of this time period, administration of PTU was stopped and rats were given one daily injection of T_3_ (20 *μ*g/100 g body mass in 0.01 N NaOH, i.p.) for three consecutive days. Animal care, maintenance, and experiments were done under the supervision of the Institutional Animal Ethics Committee (IAEC) regulated by the Committee for the Purpose of Control and Supervision of Experiments on Animals (CPCSEA), Government of India. All the chemicals used were analytical grade.

### 2.2. Tissue Processing and Isolation of Submitochondrial Fractions

Rats were sacrificed under ether anesthesia. Liver was perfused with cold 0.9% (w/v) NaCl in order to remove blood as it might interfere with the assays. The tissue was properly cleaned, freed of connective tissues, pat dried on filter paper, weighed, and stored at −80°C till further analyses. All isolation procedures were carried out at 4°C. Submitochondrial fractionation of rat liver was done as mentioned previously [11]. Protein concentration of samples was estimated following the Biuret method using bovine serum albumin as standard.

### 2.3. Evaluation of Oxidative Damages to Mitochondrial Membrane Lipids

To determine membrane lipid peroxidation (LPx), the formation of thiobarbituric acid-reactive substances (TBARS) was monitored in SMP in the presence of 0.02% (w/v) butylated hydroxytoluene to suppress artifactual peroxidation during heating [13]. Besides endogenous LPx, susceptibility of SMP to oxidants was assessed* in vitro*. For this, SMP prepared from different groups of rats were incubated without any oxidant, with H_2_O_2_ (600 nmol/mg protein) and tert-butyl hydroperoxide (600 nmol/mg protein), respectively, for 1 h at 37°C prior to TBARS determination. Only sample incubated in buffer and not exposed to any oxidant was taken as control for each set.

### 2.4. Evaluation of Oxidative Damages to Mitochondrial Proteins

Protein carbonyl content was estimated in SMP and mitochondrial matrix fraction by detecting the reaction of dinitrophenylhydrazine with protein carbonyls to form protein hydrazones [14].

### 2.5. Evaluation of Mitochondrial ROS and ROS-Metabolizing Antioxidant Enzyme (AOE) Activities

The rate of superoxide radical (O_2_^•−^) generation by SMP was measured as superoxide dismutase- (SOD-) inhibitable reduction of nitroblue tetrazolium (NBT) [11]. H_2_O_2_ content was determined in mitochondrial fraction following horseradish peroxidase-dependent oxidation of phenol red [15]. For estimating AOE activities, one milliliter of freeze-thawed mitochondrial matrix fraction was passed through 5 mL column of Sephadex G-25 in order to get rid of any interfering low molecular weight compounds. One unit of SOD activity was defined as the amount of enzyme capable of inhibiting 50% of nitrite formation under assay conditions [16]. Glutathione peroxidase (GPx) activity was assayed by measuring the rate of oxidation of NADPH in presence of hydroperoxide, reduced glutathione and glutathione reductase (GR) [17]. Total GPx activity was measured using cumene hydroperoxide as substrate whereas Se-dependent GPx was determined using tert-butyl hydroperoxide as substrate [18]. Activity of Se-independent GPx was obtained by subtracting the activity of Se-dependent GPx from that of total GPx.

### 2.6. Evaluation of Mitochondrial Protein-Bound and Nonprotein-Bound Thiol Content

For measuring protein-SH content, SMP and matrix samples were first precipitated in ice-cold 5% trichloroacetic acid (TCA) containing 0.01 N HCl and centrifuged at 1000 ×g for 15 min. Protein precipitates dissolved in 8 M guanidine hydrochloride were used to measure P-SH content, while nonprotein-SH content was estimated in TCA-treated matrix supernatants using 5,5′-dithiobis-2-nitrobenzoic acid [19, 20].

### 2.7. Evaluation of Functional Activity of Inner Mitochondrial Membrane

The activities of the mitochondrial inner membrane-associated respiratory chain enzyme complexes were assayed in SMP as detailed previously [11].

### 2.8. Histological Analyses

Following sacrifice, liver tissues were immediately fixed for histological analyses in freshly prepared sublimate formol, dehydrated in graded ethanol series, cleared in xylene, and embedded in paraffin wax. Tissues were sectioned at 5 *μ*m and the sections were stained with hematoxylin and eosin. The sections were observed under light microscope. The tissue sections were analysed by a histologist who was not aware of sample assignment to the experimental groups.

### 2.9. Detection of Apoptosis

Histochemical detection of apoptosis was performed in tissue sections prepared from liver of euthyroid, hypothyroid, and hypothyroid + T_3_ rats by terminal deoxynucleotidyl transferase- (TdT-) mediated dUTP-biotin nick end labeling (TUNEL) assay. Specific staining for* in situ* apoptosis was performed using TACS* in situ* Apoptosis Detection Kit (R&D Systems Inc., Minneapolis, USA) following the manufacturer's protocol. TUNEL positive cells were taken as the cells that were stained intense brown.

### 2.10. Statistical Analysis

Data were subjected to two-way analysis of variance followed by Duncan's new multiple range test to determine the level of significance between control and treatment means. Data were presented as mean values ± S.D. and differences between groups were considered statistically significant at the minimal level of *P* < 0.05.

## 3. Results

### 3.1. Oxidative Damages to Mitochondrial Membrane Lipids

**Figure 1:**
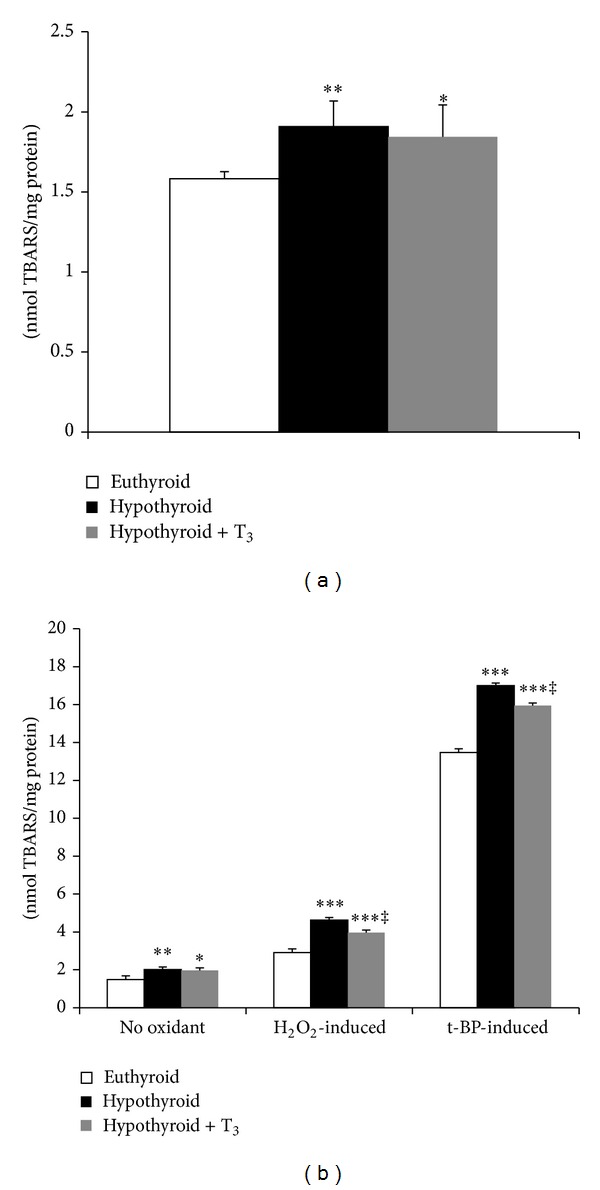
Effect of PTU-induced hypothyroidism and its reversal by T_3_ upon oxidative damage to mitochondrial membrane lipids. (a) Endogenous membrane lipid peroxidation (TBARS formed) in submitochondrial particles (SMP) isolated from liver of rats. (b)* In vitro* oxidant-induced TBARS level for which SMP were incubated without any oxidant and with H_2_O_2_ (600 nmol/mg protein) and tert-butyl hydroperoxide (600 nmol/mg protein), respectively, for 1 h at 37°C prior to TBARS determination. Data are means ± S.D. of five animals/group. Statistical significance is denoted by *P < 0.05, **P < 0.01, and ***P < 0.001 compared to euthyroid rats; ^‡^ *P* < 0.005 compared to hypothyroid rats.

Endogenous membrane LPx expressed in terms of TBARS level was significantly enhanced (20.89%, *P* < 0.01) in SMP from hypothyroid rats compared to euthyroid (Figure 1(a)). Treatment of hypothyroid rats with T_3_ reduced TBARS level from that of hypothyroid rats but could not reach the basal value. Determination of TBARS level after* in vitro* incubation with oxidants revealed that hypothyroid SMP were the most susceptible to peroxidation induced by both H_2_O_2_ and tert-butyl hydroperoxide which subsided to certain extent after T_3_ treatment (Figure 1(b)).

### 3.2. Oxidative Damages to Mitochondrial Proteins

**Figure 2:**
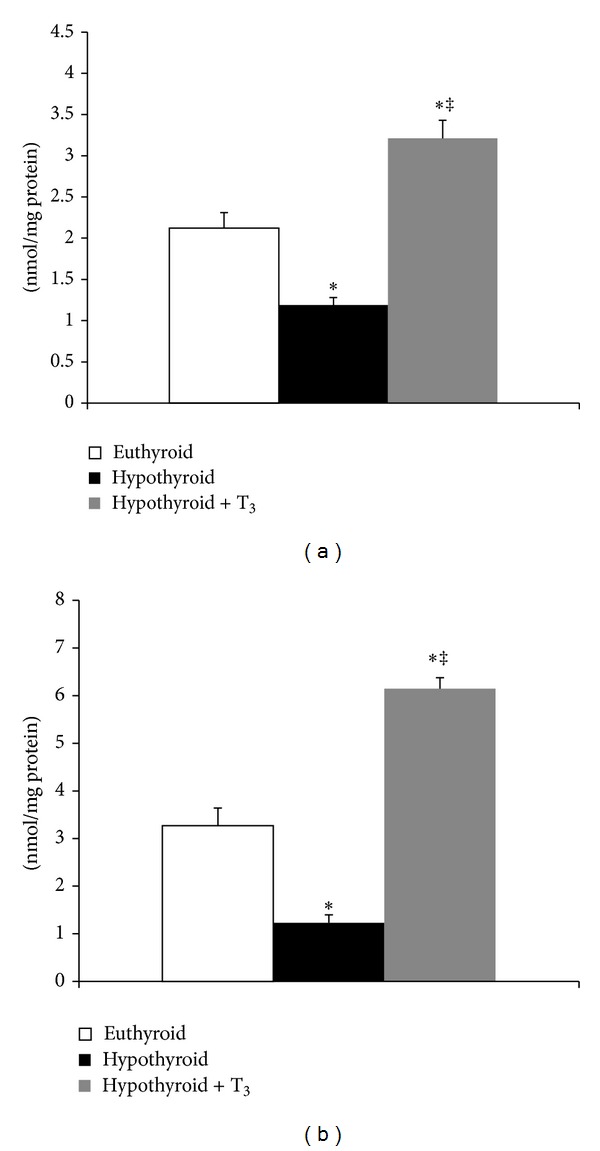
Effect of PTU-induced hypothyroidism and its reversal by T_3_ upon oxidative damage to mitochondrial proteins. (a) Protein carbonyl content in submitochondrial particles. (b) Protein carbonyl content in mitochondrial matrix fraction isolated from liver of rats. Data are means ± S.D. of five animals/group. Statistical significance is denoted by *P < 0.001 compared to euthyroid rats; ^‡^ *P* < 0.005 compared to hypothyroid rats.

Hypothyroidism caused significant reduction in protein carbonyl content in SMP (43.87%, *P* < 0.001), whereas administration of T_3_ resulted in 1.5-fold elevation compared to euthyroid (Figure 2(a)). Similar was the trend in mitochondrial matrix where hypothyroid rats showed decreased protein carbonylation (62.38%, *P* < 0.001) whereas hypothyroid + T_3_ rats recorded almost two-fold augmentation (Figure 2(b)).

### 3.3. Mitochondrial ROS and ROS-Metabolizing AOE Activities

**Figure 3:**
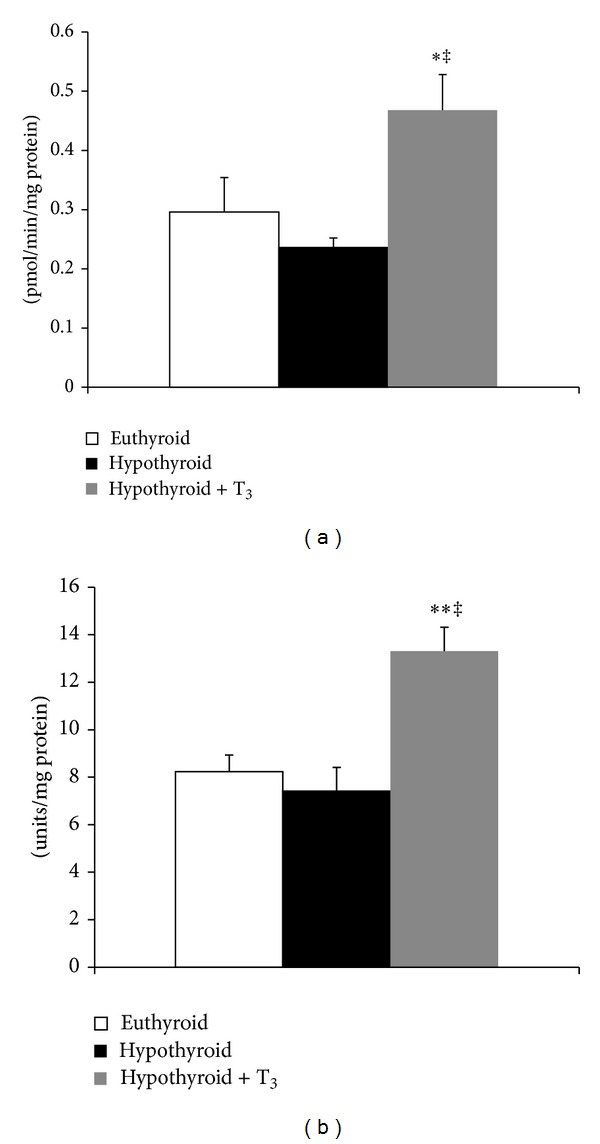
Effect of PTU-induced hypothyroidism and its reversal by T_3_ upon mitochondrial ROS and ROS-metabolizing enzymes. (a) Superoxide radical generation capacity of submitochondrial particles. (b) Superoxide dismutase activity in the mitochondrial matrix fraction isolated from liver of rats. Data are means ± S.D. of five animals/group. Statistical significance is denoted by *P < 0.005 and **P < 0.001 compared to euthyroid rats;^‡^ *P* < 0.001 compared to hypothyroid rats.

**Figure 4:**
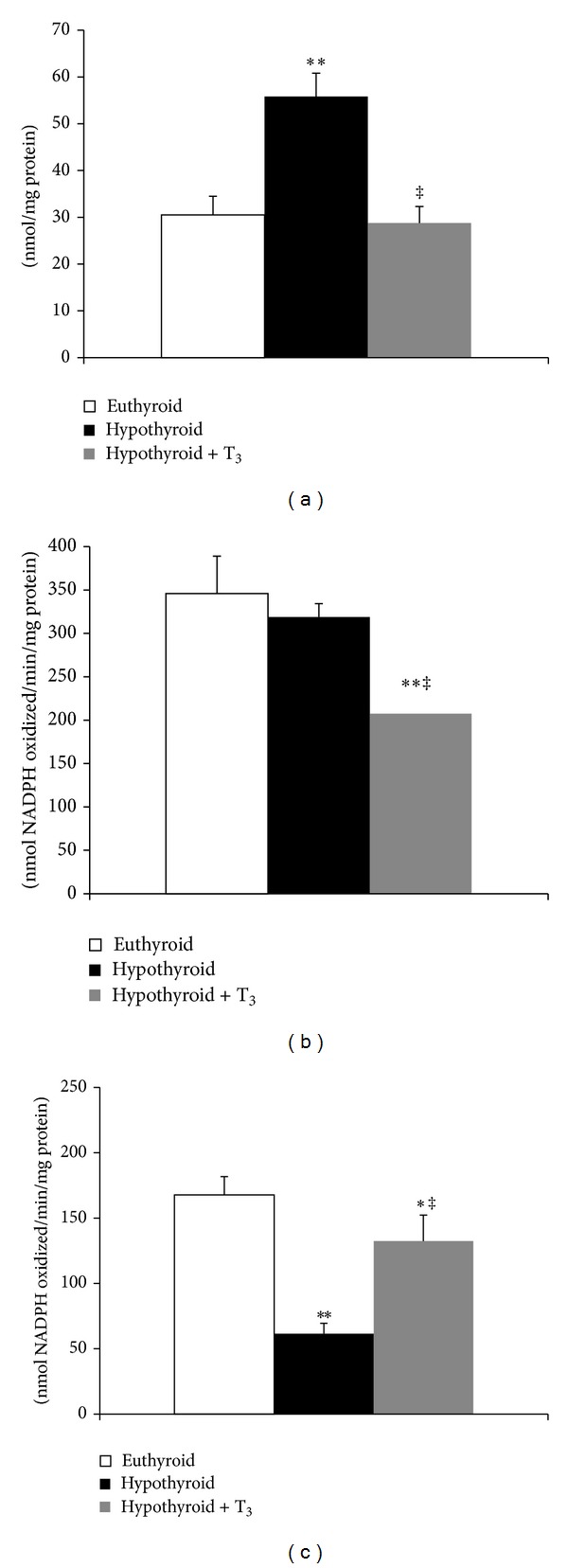
Effect of PTU-induced hypothyroidism and its reversal by T_3_ upon mitochondrial ROS and ROS-metabolizing enzymes. (a) Hydrogen peroxide content. (b) Se-dependent glutathione peroxidase (GPx) activity. (c) Se-independent GPx activity in the mitochondrial matrix fraction isolated from liver of rats. Data are means ± S.D. of five animals/group. Statistical significance is denoted by *P < 0.01 and **P < 0.001 compared to euthyroid rats; ^‡^ *P* < 0.001 compared to hypothyroid rats.

SOD-sensitive O_2_^•−^-mediated NBT reduction was significantly increased by 58.11% (*P* < 0.005) in hypothyroid + T_3_ rats (Figure 3(a)). The activity of matrix SOD was significantly enhanced (64.16%, *P* < 0.001) in T_3_-treated hypothyroid rats whereas hypothyroid rats did not record any significant change (Figure 3(b)). Hypothyroid rats exhibited almost 1.8-fold increase in H_2_O_2_ content from euthyroid (Figure 4(a)). On the other hand, hypothyroid + T_3_ rats showed H_2_O_2_ content close to basal level. While hypothyroid rats showed no change in Se-dependent GPx activity, subsequent treatment with T_3_ decreased enzymatic activity significantly (40%, *P* < 0.001) (Figure 4(b)). The trend was quite different in case of Se-independent GPx activity which was significantly downregulated in hypothyroid rats (63.33%, *P* < 0.001), whereas T_3_ treatment induced the enzyme activity albeit still lesser than euthyroid (Figure 4(c)).

### 3.4. Mitochondrial Protein-Bound and Nonprotein-Bound Thiol Content

**Figure 5:**
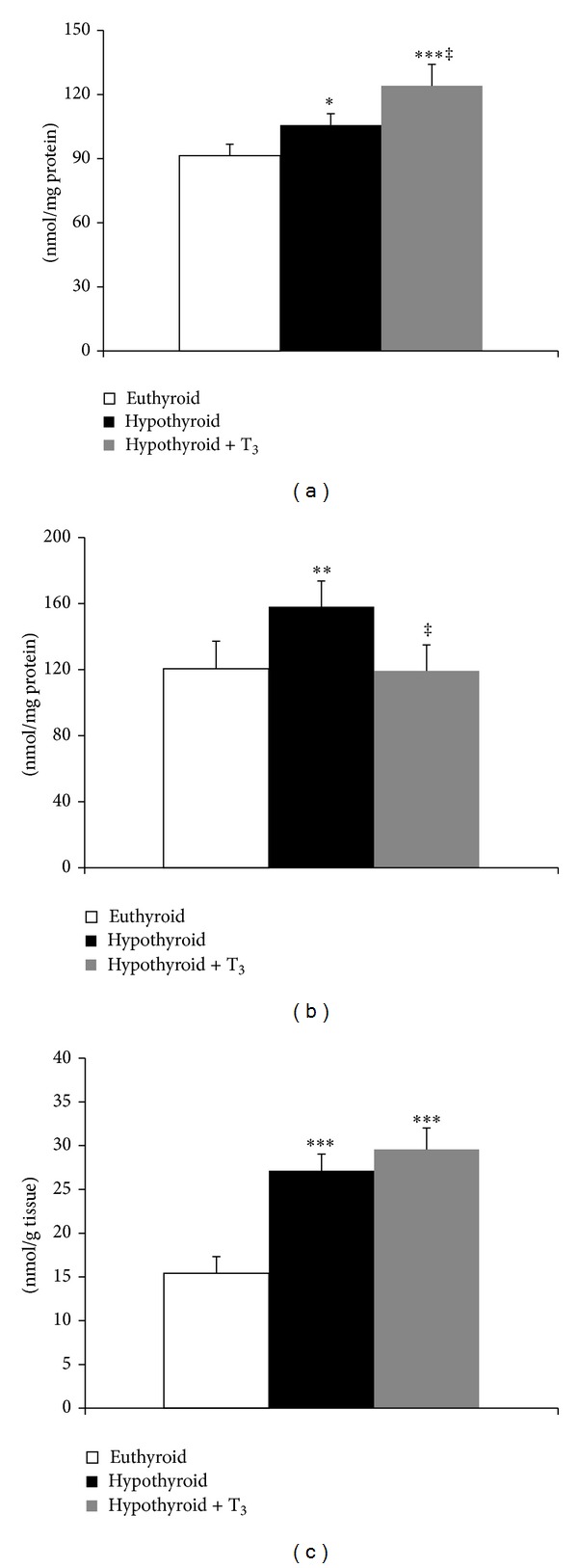
Effect of PTU-induced hypothyroidism and its reversal by T_3_ upon mitochondrial thiol content. (a) Protein-bound thiol content in submitochondrial particles. (b) Protein-bound thiol content in mitochondrial matrix fraction. (c) Nonprotein thiol content in mitochondrial matrix fraction isolated from liver of rats. Data are means ± S.D. of five animals/group. Statistical significance is denoted by *P < 0.01, **P < 0.005, and ***P < 0.001 compared to euthyroid rats; ^‡^ *P* < 0.005 compared to hypothyroid rats.

Protein-SH content was significantly enhanced in SMP and mitochondrial matrix fraction of hypothyroid rats with regard to euthyroid (Figures 5(a) and 5(b)). While administration of T_3_ to hypothyroid rats enhanced protein-SH content in SMP (35.82%, *P* < 0.001), it lowered the value to basal level in mitochondrial matrix. The nonprotein thiol content was increased significantly in both the treated groups (Figure 5(c)).

### 3.5. Functional Activity of Inner Mitochondrial Membrane

**Figure 6:**
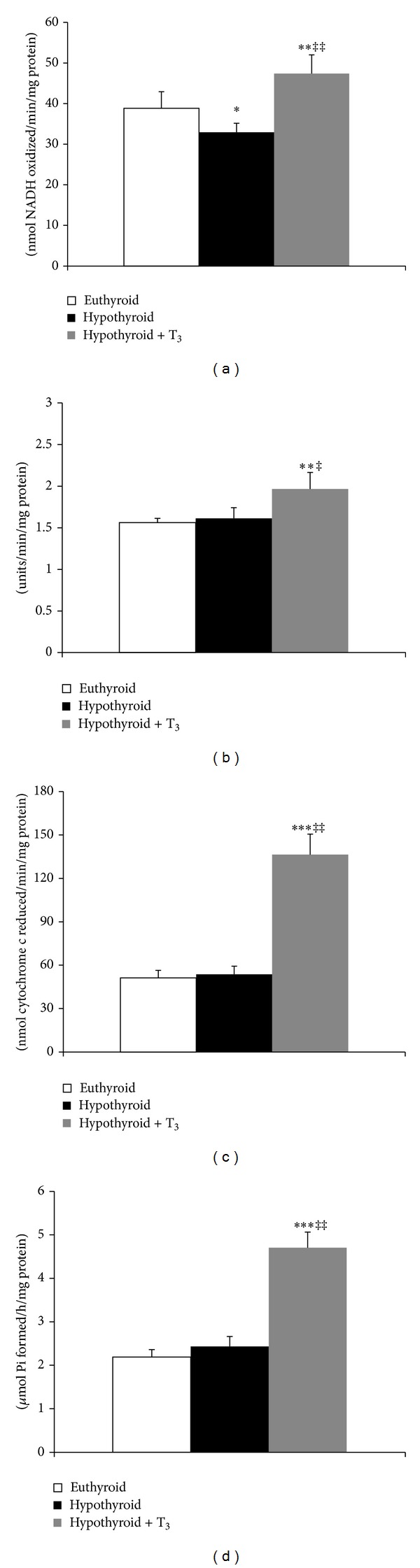
Effect of PTU-induced hypothyroidism and its reversal by T_3_ upon mitochondrial membrane-bound respiratory enzyme complexes. (a) NADH:duroquinone (DQ) oxidoreductase activity. (b) Succinate:2,6-dichlorophenolindophenol (DCPIP) oxidoreductase activity. (c) Succinate:cytochrome c oxidoreductase activity. (d) ATPase activity in the submitochondrial particles isolated from liver of rats. Data are means ± S.D. of five animals/group. Statistical significance is denoted by *P < 0.05, **P < 0.01, and ***P < 0.001 compared to euthyroid rats; ^‡^ *P* < 0.05 and ^‡‡^ *P* < 0.001 compared to hypothyroid rats.

NADH:duroquinone oxidoreductase activity was reduced by 15.18% (*P* < 0.05) in the hypothyroid rats. However, administration of T_3_ to hypothyroid rats resulted in 22.05% (*P* < 0.01) increment over euthyroid (Figure 6(a)). Succinate:DCPIP oxidoreductase activity was not affected significantly by hypothyroid condition. However, treatment of hypothyroid rats with T_3_ caused 26.28% (*P* < 0.01) enhancement in enzymatic activity (Figure 6(b)). Administration of T_3_ caused 2.7-fold increase in succinate:cytochrome c oxidoreductase activity (Figure 6(c)). ATPase activity was significantly enhanced (*P* < 0.001) in hypothyroid rats administered with T_3_ (Figure 6(d)).

### 3.6. Histological Analyses and Detection of Apoptosis

**Figure 7:**
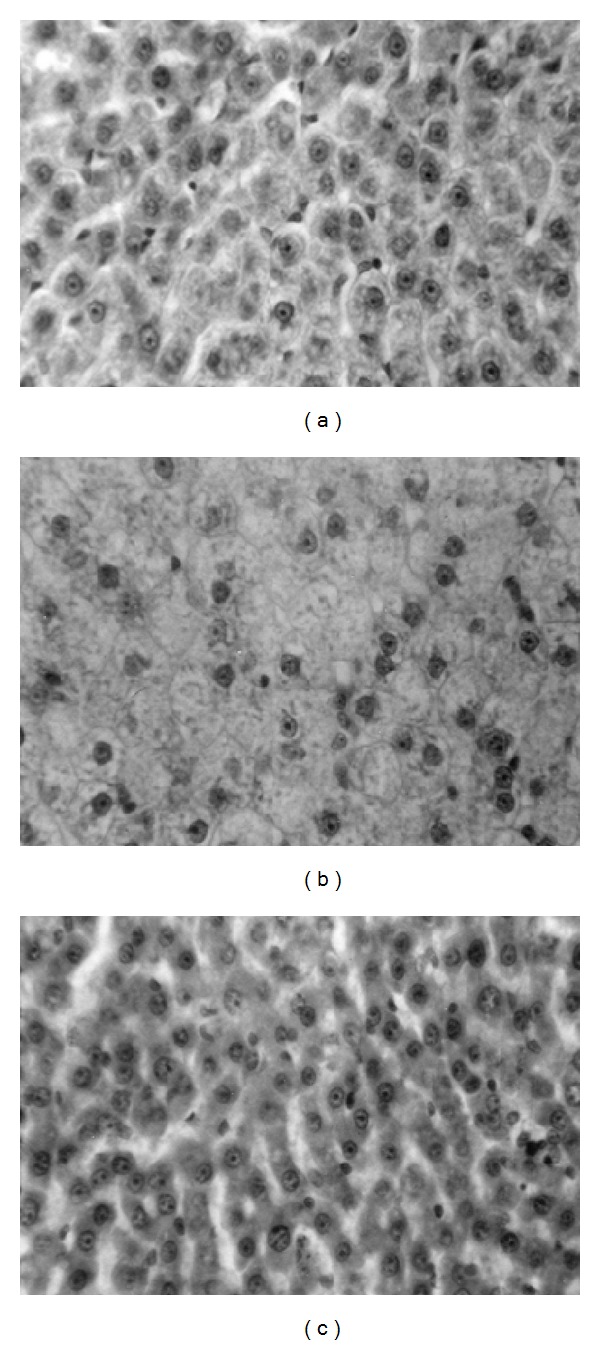
Photomicrographs of transverse sections of liver from (a) euthyroid rats, (b) hypothyroid rats, and (c) hypothyroid + T_3_ rats. Haematoxylin and eosin stain (400x).

**Figure 8:**
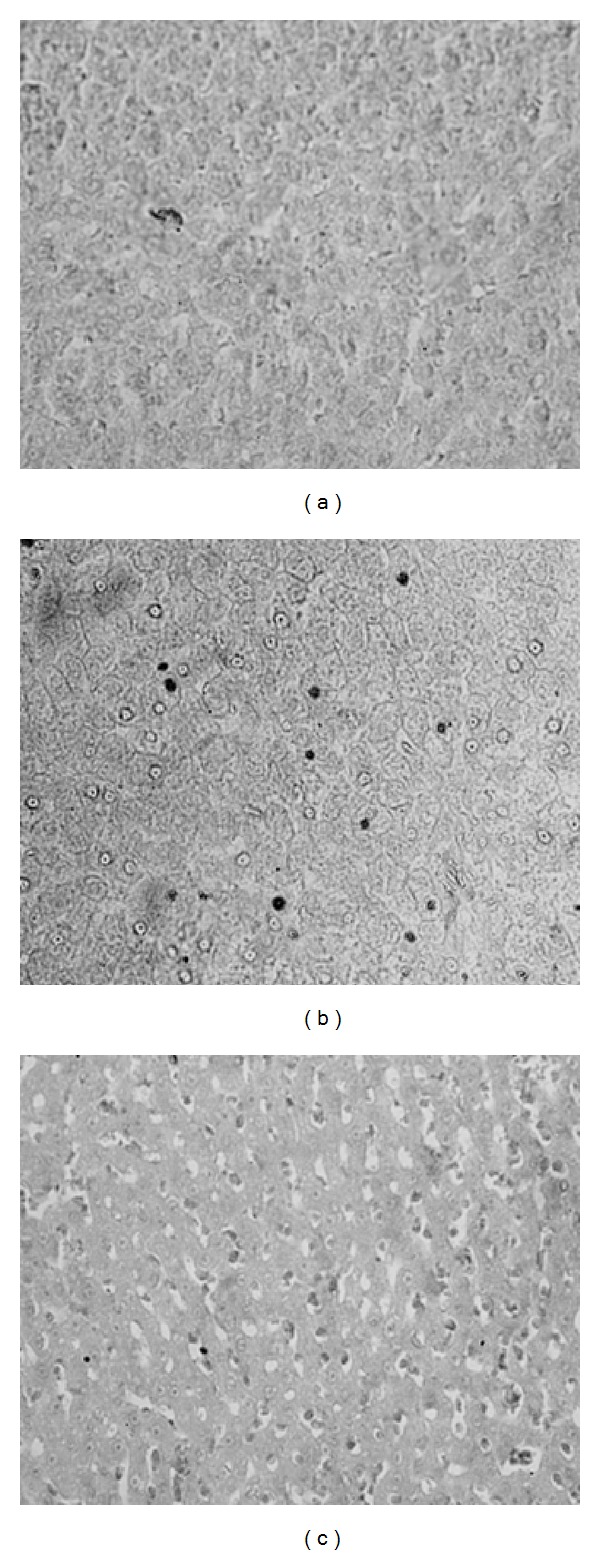
Photomicrographs of transverse sections of liver from (a) euthyroid rats, (b) hypothyroid rats, and (c) hypothyroid + T_3_ rats showing the results of TUNEL assay at 400x.

Euthyroid animals displayed normal histoarchitecture of rat liver. However, liver sections from hypothyroid rats showed marked reduction in the number of cells apart from disappearance and disintegration of the nuclei in several cells. On the other hand, administration of T_3_ to hypothyroid rats clearly revealed hepatocyte proliferation (Figures 7(a)–7(c)). Liver sections from hypothyroid rats exhibited considerable presence of TUNEL positive cells, indicating onset of apoptosis (Figures 8(a)–8(c)). However, hypothyroid + T_3_ rats showed opposite effect since the sections responded negatively to the TUNEL assay.

## 4. Discussion

Hypothyroidism is known to decrease mitochondrial oxygen consumption and ATP synthesis [21] while injection of T_3_ increases oxygen consumption and metabolic rate [22]. It was later revealed that mitochondrial production of ROS mostly occurs during state IV respiration [23], particularly in the proximity of complex I and complex III of the electron transport chain [24]. Presently, the analyses of the activities of mitochondrial inner membrane-bound enzyme complexes involved in cellular respiration revealed certain interesting aspects. Hypothyroid condition did not alter activities of respiratory chain complexes except that it had a strong influence on NADH-DQ oxidoreductase activity which was significantly downregulated. In stark contrast, T_3_ supplemented hypothyroid rats exhibited substantially increased activities of all enzyme complexes in liver SMP. Unfortunately, cytochrome c oxidase activity could not be estimated in this study. It was earlier reported that mRNAs and protein levels of subunits of cytochrome c oxidase in liver were downregulated or unaffected in hypothyroid state, but, after thyroid hormone treatment, the levels of gene products increased rapidly reaching supranormal levels [25]. In fact, injection of T_3_ into euthyroid rats accentuated activities of respiratory chain complexes in liver mitochondrial fraction [26] and SMP [11]. It is apparent from this study that although induction of hypothyroidism did not register any significant change in ATPase activity, subsequent treatment with T_3_ caused significant enhancement. Vacca et al. [27] reported impaired transport of phosphate and adenine nucleotide across the inner mitochondrial membrane in hypothyroidism that was accompanied with decreased gene expression of adenine nucleotide translocase. Therefore, while T_3_ upregulates all the respiratory chain complexes of inner mitochondrial membrane accounting for enhanced oxygen consumption and metabolic rate, complex I is a vulnerable target in hypothyroid condition. This is substantiated by the fact that hypothyroid phenotype is contributed by mitochondrial complex I inactivation due to translocated neuronal nitric oxide synthase [28]. Nevertheless, in both the altered thyroid states, electron leakage and generation of ROS are an unavoidable consequence.

We measured O_2_^•−^ generation capacity of inner mitochondrial membrane using SMP that are devoid of SOD. T_3_-induced increase in O_2_^•−^ generation is well known [11, 29, 30]. SOD is the primary AOE that dismutates O_2_^•−^. Mitochondrial matrix contains MnSOD which is encoded by nuclear gene. Its activity was recorded to be high in response to T_3_ treatment. This is accounted by the fact that T_3_ can upregulate MnSOD expression through NF-*κ*B activation [31]. Mitochondria are effective sources of H_2_O_2_ primarily through SOD and outer membrane enzyme monoamine oxidase. Besides, H_2_O_2_ is generated in cytosol by several oxidases. Since H_2_O_2_ can easily traverse membranes and can access distant sites, therefore, we performed H_2_O_2_ assay in mitochondrial fraction in order to get an idea about total mitochondrial H_2_O_2_ content. Higher H_2_O_2_ levels in hypothyroid mitochondria could arise due to ineffective function of H_2_O_2_-metabolizing AOEs as reflected in diminished total GPx activity. When GPx isoenzyme activities in response to treatment were analyzed, it was observed that Se-independent GPx activity was reduced in response to hypothyroidism but was enhanced when challenged with T_3_. On the contrary, Se-dependent GPx activity was significantly reduced when hypothyroid rats were challenged with T_3_. Thus, these results clearly establish a differential regulatory influence of T_3_ on expression of different isoenzymes of GPx.

Both oxygen consumption and free radical production take place in mitochondrial inner membrane. The sensitivity of these membranes to oxidative damage is strongly dependent on their unsaturated fatty acid content. The lipid environment can directly affect membrane function, including mitochondrial electron transport and ROS production [32]. Available data on lipid peroxidative processes and oxidative status in hypothyroid liver are controversial. It was reported that levels of LPx products in liver from hypothyroid rats [33] and mice [21] do not differ from euthyroid values. On the other hand, Mogulkoc et al. [34] reported reduced MDA level in hepatic tissue from hypothyroid rats. It was asserted that hypothyroidism provided protection against LPx [35]. It further prevented increase in LPx as well as tissue damage induced by intracolonic administration of trinitrobenzene sulfonic acid and decreased susceptibility to oxygen radical-induced lung damage in newborn rats exposed to prolonged hyperoxia [36, 37]. The lower toxicity of arsenic in hypothyroid animals was associated with prevention of arsenic-induced LPx in liver and kidneys [38]. Furthermore, hypothyroidism was able to protect against acetaminophen hepatotoxicity [39]. Serum TBARS concentration did not show differences between euthyroid, overt hypothyroid, and subclinical hypothyroid women [40]. On the contrary, hypothyroidism did not provide protection from LPx in testicular mitochondria as evident from augmented TBARS levels in SMP [12]. There is also evidence of increased LPx in erythrocytes, heart, and plasma of PTU-treated rats [41, 42] and serum of hypothyroid patients [43–45]. In conformity with this, the present study revealed greater extent of mitochondrial membrane lipid damage in hypothyroidism which was alleviated upon T_3_ treatment. A similar adaptive response was reported in hyperthyroid mice arising from decreased fatty acid unsaturation index [21]. In a study in hypothyroid patients before and after treatment, Baskol et al. [46] reported that MDA levels were higher in patients with hypothyroidism before treatment, which subsided after thyroxine replacement. It seems that lack of effective antioxidant protection as well as possible prooxidant activities could explain the higher than normal LPx levels. Se-independent GPx is known to catalyze GSH-dependent reduction of phospholipid hydroperoxides* in situ *in biological membranes [47], and its overexpression in cells attenuates LPx under normal condition as well as during oxidative stress. Thus, reduction in Se-independent GPx activity in hypothyroid rats might be the reason for observing significantly high TBARS level as well. Although injection of T_3_ induced the activity, it could not restore the normal level. These data clearly establish the pivotal role essayed by Se-independent GPx in maintaining mitochondrial membrane integrity in thyroid dysfunction.

Quite intriguingly, our results indicate that hypothyroid state is accompanied with general decrease in oxidatively damaged mitochondrial proteins. Although lipid peroxidation end products such as malondialdehyde and 4-hydroxynonenal are known to initiate carbonylation of proteins, yet these parameters may not always be correlating with each other. It is important to consider that the steady state level of proteins in cells is dependent on both synthesis and degradation, that is, metabolic turnover. Accumulation of altered proteins in tissues can be either due to a higher rate of oxidation and other types of modifications, due to a lower rate of degradation of the modified proteins, or due to both. Oxidative modification of a protein makes it more susceptible to proteolysis largely due to the unfolding of the targeted protein domains. Unfolding results in an increased exposure of hydrophobic residues that are normally hidden in the interior of soluble proteins and such hydrophobic patches are known to favour recognition and degradation by the proteasome and the Lon protease [48, 49]. In addition, lipid oxidation is the primary event following oxidative stress, whereas protein carbonylation occurs secondarily. On the other hand, hypothyroid + T_3_ rats showed higher index of oxidative damage to proteins in both SMP and matrix fractions. Significantly higher protein hydrazone derivatives were seen in these rats. Stability of protein-SH groups was also adversely affected in the mitochondrial matrix fraction. This provides definitive evidence that T_3_ renders the proteins of the mitochondrial subcompartments more vulnerable to oxidative damage and the utilization of ROS in lipid and protein oxidation of SMP is reciprocal in altered thyroid states.

Glutathione (GSH), the most predominant nonprotein thiol, is involved in many biological activities including neutralization of ROS, detoxification of xenobiotics, and maintenance of SH level in proteins [50]. Effect of hypothyroidism on hepatic GSH is controversial. As observed presently, increased nonprotein thiol content in hypothyroid condition or in case of T_3_ supplementation might be an adaptive response to protect mitochondria against oxidative damage. In fact, T_3_ is known to trigger GSH biosynthesis [51]. However, in hypothyroid condition, GR activation contributes to the GSH pool by rapid conversion of GSSG into GSH. Increased protein-SH signifies proteins to be in their native state while its decrease suggests oxidative predominance.

Histological analyses of liver samples interestingly showed recovery of cellular integrity in hypothyroid rats supplemented with T_3_. This finding is well justified by the fact that T_3_ powerfully induced hepatocytes to enter S phase 24 h after injection [52]. T_3_-induced cyclin D1 expression enhanced phosphorylation of pRb and increased expression of transcription factor E2F mediated hepatocyte proliferation [53]. Besides, Alisi et al. [54] found that, both in control and in partially hepatectomized animals, hyperthyroidism increased cyclins D1, E, and A levels and the activity of cyclin-cdk complexes and decreased the levels of cdk inhibitors such as p16 and p27. These authors further showed that hypothyroidism caused downregulation of the activity of cyclin-cdk complexes decreasing cyclin levels. Thus, T_3_ exerts proproliferative effects on liver cells by regulating cell cycle proteins.

Morphological hallmarks of apoptosis in the nucleus are chromatin condensation and nuclear fragmentation [55]. The condensation starts peripherally along the nuclear membrane, forming a crescent or ring-like structure. During later stages of apoptosis the nucleus further condenses, and finally it breaks up inside a cell with an intact cell membrane, a feature described as karyorrhexis [56]. DNA strand breaks can be detected by incorporating labeled dUTP by the TUNEL method. The presence of apoptotic cells in hypothyroid liver sections as revealed from TUNEL assay clearly indicates activation of apoptotic pathways, which was later reversed upon administration of T_3_. It is suggested that hypothyroidism-induced cytochrome c release to cytosol during early development might contribute to initiation of apoptosis through formation of apoptosomes and activation of caspase cascade. Moreover, congenital hypothyroidism increased not only the extent but also the duration of apoptosis following similar mitochondrial mechanism in hippocampal neurons of developing rats [57]. The antiapoptotic effects of T_3_ are well supported by both* in vivo* and* in vitro* reports. Fernández et al. [31] showed that administration of 0.1 mg T_3_/kg body weight for 68–72 h caused upregulation in expression of the antiapoptotic protein Bcl-2. T_3_ inhibited TNF*α*/Fas-induced apoptosis in mouse hepatocytes [58]. It is relevant to mention in this context that T_3_ protected cardiac myocytes against ischemia-induced apoptosis via Akt signaling [59]. Thus, T_3_ can initiate hepatoprotective mechanism in pathological conditions by suppressing apoptosis and enabling hepatocyte survival.

## 5. Conclusion

It is apparent from the above data that hypothyroidism brought about metabolic suppression antagonistic to the hypermetabolic state induced by T_3_, yet it inflicted considerable cellular injury primarily through oxidative damage to mitochondrial membrane lipids and inhibition of the electron transport chain complex I. Hydroperoxide neutralization was severely affected in hypothyroidism and the compromised antioxidant defenses failed to contain the high levels of ROS. Accumulation of ROS and loss of membrane integrity are the two primary factors that potentially trigger apoptosis. However, T_3_ treatment exerted antiapoptotic and proproliferative effects on hypothyroid liver cells. Nevertheless, mitochondrial membrane and matrix proteins seemed more vulnerable in hyperthyroid state, which also disturbed the mitochondrial oxidant  :  antioxidant balance. Quite aptly, a recent clinical study suggested that antioxidant therapy should be advised along with thyroid hormone replacement therapy to diminish further complications [60]. Taken together, these data confirm that hypothyroidism-induced severe hepatocellular injury can be substantially recovered upon T_3_ supplementation.
